# Ultra-Broadband Solar Absorber and High-Efficiency Thermal Emitter from UV to Mid-Infrared Spectrum

**DOI:** 10.3390/mi14050985

**Published:** 2023-04-30

**Authors:** Fuyan Wu, Pengcheng Shi, Zao Yi, Hailiang Li, Yougen Yi

**Affiliations:** 1 Joint Laboratory for Extreme Conditions Matter Properties, Southwest University of Science and Technology, Mianyang 621010, China; 15284169398@163.com (F.W.); pengchengshi114@163.com (P.S.); 2 School of Chemistry and Chemical Engineering, Jishou University, Jishou 416000, China; 3 Key Laboratory of Microelectronic Devices & Integrated Technology, Institute of Microelectronics, Chinese Academy of Sciences, Beijing 100029, China; lihailiang@ime.ac.cn; 4 College of Physics and Electronics, Central South University, Changsha 410083, China; yougenyi@csu.edu.cn

**Keywords:** ultra-wideband absorption, high thermal radiation efficiency, metal-dielectric-metal composite structure, heat emitter

## Abstract

Solar energy is currently a very popular energy source because it is both clean and renewable. As a result, one of the main areas of research now is the investigation of solar absorbers with broad spectrum and high absorption efficiency. In this study, we create an absorber by superimposing three periodic Ti-Al_2_O_3_-Ti discs on a W-Ti-Al_2_O_3_ composite film structure. We evaluated the incident angle, structural components, and electromagnetic field distribution using the finite difference in time domain (FDTD) method in order to investigate the physical process by which the model achieves broadband absorption. We find that distinct wavelengths of tuned or resonant absorption may be produced by the Ti disk array and Al_2_O_3_ through near-field coupling, cavity-mode coupling, and plasmon resonance, all of which can effectively widen the absorption bandwidth. The findings indicate that the solar absorber’s average absorption efficiency can range from 95.8% to 96% over the entire band range of 200 to 3100 nm, with the absorption bandwidth of 2811 nm (244–3055 nm) having the highest absorption rate. Additionally, the absorber only contains tungsten (W), titanium (Ti), and alumina (Al_2_O_3_), three materials with high melting points, which offers a strong assurance for the absorber’s thermal stability. It also has a very high thermal radiation intensity, reaching a high radiation efficiency of 94.4% at 1000 K, and a weighted average absorption efficiency of 98.3% at AM1.5. Additionally, the incidence angle insensitivity of our suggested solar absorber is good (0–60°) and polarization independence is good (0–90°). These benefits enable a wide range of solar thermal photovoltaic applications for our absorber and offer numerous design options for the ideal absorber.

## 1. Introduction

In the recent past, people have been using more and more energy sources, traditional fossil energy sources have been in short supply, and people are exploring more and more renewable energy sources; therefore, as a new renewable clean energy, solar energy has become the focus of attention [1,2,3]. There has been extensive research on clean energy sources including solar energy to solve the energy shortage problem. However, despite the extensive research on solar absorbers, there are still many shortcomings. For example, the absorption band width is narrow, the absorption intensity is not high, and the structure is complicated, which limits the application of absorbers in solar photovoltaic and other fields [4,5,6,7,8]. Therefore, it is important to explore a wideband absorber with good oblique incidence characteristics and polarization angle independence and high thermal radiation efficiency. Meanwhile, ultra-wideband absorbers based on refractory materials have great application value because thermophotovoltaic devices need to work in high-temperature environments.

The absorber’s ability to absorb has been significantly impacted by various model structures. Landy first proposed metal-insulator-metal frameworks for narrowband ideal absorbers in 2008 [9], and the search for perfect absorbers has become the focus of many researchers, but early metamaterials were designed to achieve single-band or multi-band absorption [10,11,12]. At present, there are two methods to achieve broadband absorption in metal nanostructure absorbers. One is to introduce multiple different nanoresonators into the unit structure of metamaterial. These resonators’ ability to create many absorption peaks at various spectral frequencies enables them to accomplish broadband absorption; nevertheless, their complicated system and demanding technical specifications are disadvantages [13]. Adding composite films made of metal dielectric layers on top of one another is another method for achieving broadband absorption. This absorber is not angle-sensitive and can achieve mid-infrared band broadband absorption [14]. In 2018, Hu et al. created a continuous eight-layer metal dielectric film packing arrangement in order to achieve nearly perfect absorption in the range of 250 to 2000 nm [15]. Despite great absorption efficiencies in both instances, there is little NIR band absorption.

The efficiency of the absorber in terms of absorbing energy is also strongly influenced by the difference in materials. Solar absorber studies frequently make use of precious metals such as Ag, Au, and Cu. Although good absorption can be obtained, the cost is high and absorbers designed with precious metal materials are more prone to deformation at high temperatures. The structures based on precious metal absorber materials can also be deformed under strong light irradiation and lose their original absorption properties [16]. Meanwhile, titanium and tungsten are widely used as refractory metals and alumina. In 2015, using a metal-dielectric composite structure as the foundation, Ding et al. suggested a film-stacked absorber [17], where metallic material contains the high-temperature-resistant metal Ti. Simulations and experiments confirmed its high absorptivity, omnidirectivity, and polarization independence in the whole visible band. For the wavelength range of 400 nm to 900 nm, the estimated absorption rate is greater than 90%, with an average absorption rate of 96.03%. In the same year, similar plane cell layer stacking construction with a 99.5% average absorption rate in the 400–800 nm spectral range was proposed by Li et al. [18]. We conclude that the high-temperature refractory-based light absorber has great prospects for this application.

Thermal radiation refers to the electromagnetic waves emitted by objects into the surrounding space at different temperatures. This radiation is produced by the thermal motion of molecules inside an object, so it is also called thermal motion radiation. There are four important laws about thermal radiation: Kirchhoff’s radiation law, Planck’s radiation distribution law, Stefan–Boltzmann law, and Wien’s displacement law. The wavelength and intensity of thermal radiation are related to the temperature of the object, and the higher the temperature, the shorter and greater the intensity of the radiation. Thermal radiation has a wide range of applications in daily life, such as infrared thermal imagers, energy conversion in solar panels, thermal radiation temperature measurement and so on [19,20]. As an important physical phenomenon, thermal radiation has a wide range of applications in daily life and various fields. Through the research and application of heat radiation, we can make better use of natural resources, improve production efficiency, and ensure the safety of human life. In this paper, a three-layer periodic Ti-Al_2_O_3_-Ti disk structure is proposed on a W-Ti-Al_2_O_3_ composite nanofilm, and the multilayer nanodisk structure is easy to provide a near-field coupling effect and improve the absorption efficiency. The structure has an average absorption efficiency of 95.8% and an absorption bandwidth of 2811 nm. More notably, this structure has a high thermal radiation efficiency of 94.4% at 1000 K. This also provides new ideas for the application breakthrough of solar absorbers.

High strength, excellent heat and corrosion resistance, excellent ductility, and low density are all characteristics of titanium [21]. As a refractory metal, titanium has a high melting point (1668 °C), good stability, and resistance to magnetization under strong magnetic fields [22]. The most important advantages of tungsten as a refractory metal are its good high-temperature strength (melting point is about 3410 °C) and very stable chemistry [23]. The absorber has good stability, its cost is lower than that of precious metals, and compared with other expensive metals Au and Ag, Ti as a resonant metal can excite a wider bandwidth response in the infrared band, and it is easier to achieve perfect absorption in the ultra-wideband [24,25,26].

Through the aforementioned examples, it was discovered that, despite the fact that some of the proposed broadband absorbers contain refractory metallic materials, others also contain noble metals, which lack benefits in terms of thermal stability in addition to being expensive, and the absorption bandwidth typically does not reach 2000 nm [27]. We proposed a three-layer periodic Ti-Al_2_O_3_-Ti disc structure overlaid on a W-Ti-Al_2_O_3_ composite film as an ultra-broadband solar absorber to address the aforementioned set of issues. The bandwidth of this MIM composite structure is effectively extended [28], and the average absorption efficiency in the entire wavelength range of 200–3100 nm is as high as 95.8%. The absorption efficiency is better than 90% up to 2811 nm. The three high melting point materials utilized in the model offer a solid assurance for the thermal stability of the absorber. Additionally, the absorber has a very high thermal radiation intensity, with a weighted average absorption efficiency of 98.3% (AM1.5) and a radiation efficiency of over 90% at 1000 K. As a result, when compared to other absorbers, our proposed absorber has excellent potential for use in the field of energy harvesting technologies, such as high absorption, broadband, and high-temperature-resistant metal-dielectric composite structures and solar thermal photovoltaics.

## 2. Structure and Design

**Figure 1:**
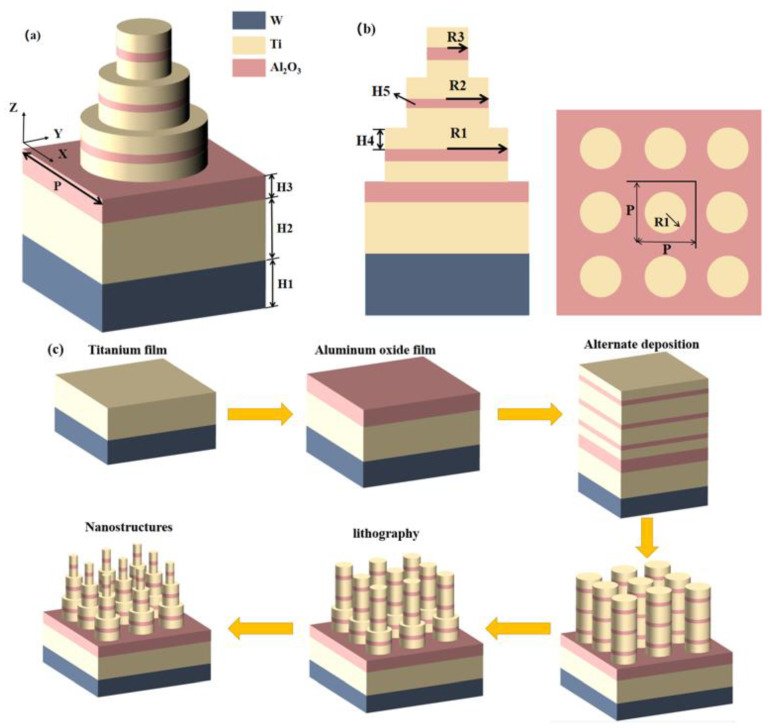
(**a**) An illustration of the perfect absorber in 3D. (**b**) The ideal absorber’s XOY and XOZ plans. (**c**) A tangible illustration of the manufacturing procedure for the ideal absorber.

We model a stacked cubic solar absorber made of metal and dielectric materials using the FDTD technique [29,30]. In order to obtain high simulation accuracy, we chose the Al_2_O_3_ as the dielectric layer in the FDTD algorithm and determined the dielectric constants of Ti and W using experimental data of Palik [31]. In this study, we propose an absorber structure, as shown in Figure 1a, is made up of three Ti-Al_2_O_3_-Ti nanosheets of varying radii and W-Al_2_O_3_-Ti films. We employ the refractory material W, which melts at 3420 °C, and the refractory metal Ti, which melts at 1668 °C. The structure at high-temperature work is unaffected since the dielectric layer, Al_2_O_3_, is also an insoluble substance at high temperatures.

We used a plane wave with an incidence wavelength of 200–3100 nm that was parallel to the *x*-axis in the simulation computation. With a grid precision of 2, periodic boundary conditions, an endlessly distributed periodic array in the *x* and *y* directions, a perfectly matched PML layer in the *z* direction, and a light source incident in the opposite direction of the *z* axis; we used these techniques in our simulation model. The absolute symmetry of the absorber renders it polarization insensitive. Our developed structures have heights of H1 = 220 nm (W), H2 = 240 nm (Ti), and H3 = 50 nm (Al_2_O_3_). The metal thickness of the composite disk structure is H4 = 45 nm and the dielectric thickness is H5 = 30 nm. Our absorbing disk has a radius of R1 = 160 nm, R2 = 120 nm, and R3 = 80 nm, and its structural period is P = 400 nm, as shown in Figure 1a,b. The value of the light absorption A(ω) is equal to A = 1 − T(ω) − R(ω) [32,33,34,35], and since our substrate material is an opaque material W, the transmittance T is completely cancelled, indicating that the spectrum T(ω) = 0. Therefore, the absorption A(ω) can be obtained by 1 − R(ω) [36]. Figure 1c shows a schematic diagram of the preparation process and flow of the solar absorber. In preparing the solar absorber, we can first deposit Ti (240 nm), Al_2_O_3_ (50 nm) thin film by ion beam sputtering on the surface of W (220 nm) substrate, and then deposit Ti (45 nm) - Al_2_O_3_ (50 nm) - Ti (45 nm) composite thin film structure by magnetron sputtering. Finally, the desired microstructure can be obtained by photolithography and electron beam evaporation.

## 3. Results and Discussion

**Figure 2:**
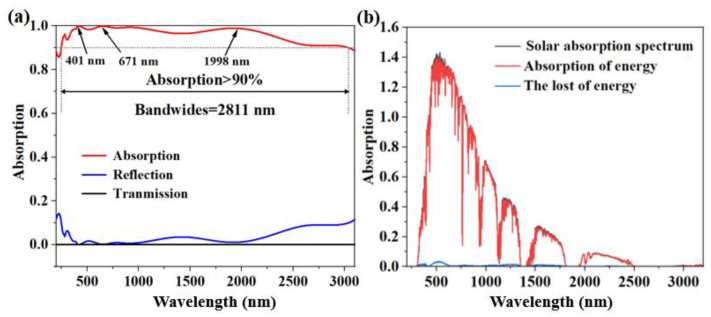
(**a**) Under typical incoming light, the spectrum of reflection, absorption, and transmission. (**b**) A plot of AM1.5’s solar absorption and loss in the 200–3100 nm wavelength range.

Planar light’s incident light line was chosen to be in the 200–3100 nm range. Figure 2 displays the simulation findings from this effort. Figure 2a displayed the simulation’s outcomes. The bandwidth is 2811 nm in the wavelength range of 244–3055 nm, and the absorption rate is better than 90%. From 200 nm to 3100 nm, the average absorption rate is 95.8%, and the NIR absorption loss after 3055 nm is primarily focused in the NIR area. In order to conduct the investigation, we chose three bands with a high absorption intensity, so as to facilitate the subsequent analysis of why we can obtain such good results. λ = 401 nm, 671 nm, and 1988 nm, were selected, and their absorption intensity arrived at 99.88%, 99.80%, and 97.64%, respectively.

The spectrum absorption coefficient is an important metric to assess a solar absorber’s capacity to capture heat [37]. Equation (1) is the formula for the entire solar energy spectrum incident at AM1.5 [38].

(1) $$\eta_{A}=\frac{\int_{\lambda_{\min}}^{\lambda_{\max}}A\left(\omega \right)\cdot I_{AM1.5}\left(\omega \right)d\omega }{\int_{\lambda_{\min}}^{\lambda_{\max}}I_{AM1.5}\left(\omega \right)d\omega }$$

where I_be_(ω, T) is the intensity of frequency ω and temperature T of the ideal blackbody optical spectrum. In comparison to the ideal blackbody model, the thermal emitter exhibits almost perfect emission intensity in the wavelength range of no more than 3100 nm for this solar absorption system at a temperature of 1000 K. A new method of realizing blackbody thermal emission or light source is made possible by the thermal emitter’s up to 94% emission efficiency in the 280–3100 nm spectrum. Where I_be_(ω, T) is the intensity of the ideal blackbody optical spectrum at frequency ω and temperature T [39]. To be able to highly match the solar radiation, we extend the spectral range from the ultraviolet to the infrared range. The minimum (λ_min_) and maximum (λ_max_) wavelengths are 200 nm and 3100 nm, respectively, and the thermal emitter exhibits almost perfect emission intensity in the wavelength range up to 3100 nm in this solar absorption system at a temperature of 1000 K compared to the ideal blackbody model. The emission efficiency of the thermal emitter of up to 94% in the spectrum of 280–3100 nm makes it possible to realize new methods of thermal emission from black bodies or light sources. Using previously published works on solar absorber [40,41,42,43,44], Table 1 provides a comparison of their performance. Compared with these solar absorbers, we can see that the proposed structure has better performance.

**Figure 3:**
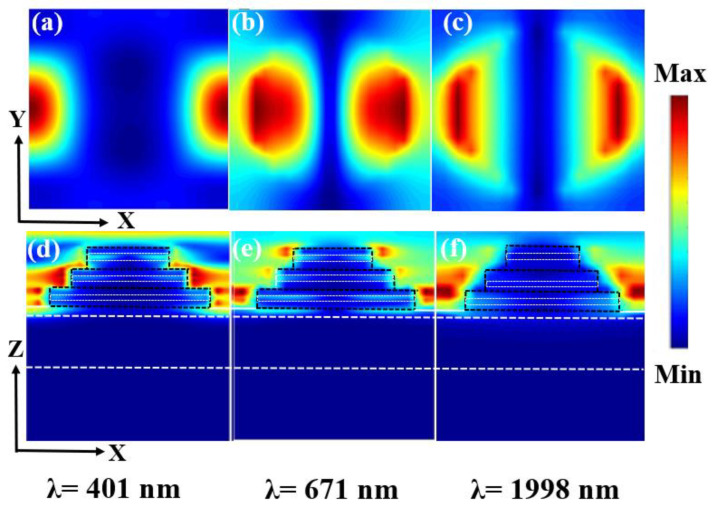
(**a**–**c**) Three absorption peaks are distributed across an electric field in the XOY plane. (**d**–**f**) Three absorption peaks on the XOZ plane′s electric field distribution.

In addition to calculating and examining the physical mechanism underlying solar absorber broadband absorption, this study also makes an educated guess regarding the distribution of electric field intensities among these three absorption peaks. The electric field strengths of the three absorption peaks’ XOY and XOZ cross sections are shown in Figure 3. When λ = 401 nm, as depicted in Figure 3a,d, the majority of the electric field is concentrated on the sidewalls of the nanodisk, and the space between the absorber units can be thought of as resembling a cavity structure. Under the action of incident light, cavity film resonance is formed in the cavity. Due to the formation of resonances in the cavity membrane, the energy is bound in the cavity, significantly enhancing the electric field there [45,46]. Therefore, it is reasonable to suppose that oscillations outside of the structure, at which the incident light interacts between adjacent disks through the excited cavity modes at this wavelength, are the primary cause of the absorption in the near-UV region. The connection gap region between the Ti and Al_2_O_3_ films and the angular surface region of the perfect absorber nanodisk array have significant electric fields at 671 nm, and plasmon resonance also occurs at this wavelength. When the wavelength is 1998 nm, according to Figure 3c, the surface plasmon intensity rises (f). Due to the incident light’s growing wavelength, as shown in Figure 3c,f, Al_2_O_3_’s plasmon resonance at 1998 nm is more excited. Excitation cavity coupling was performed for films with R = 160 nm, 120 nm, and 80 nm, while high plasmon resonances were found around the nanodiscs of the MIM structure. The discs with different radii and their near-field coupling caused strong absorption in the near-infrared band. We can, therefore, conclude that the ultra-wideband absorption effect, which can be produced by excitation of various wavelength harmonic oscillator absorption or tuning absorption, can be created via the cavity mode coupling of the structure, the near-field coupling, and the plasmon resonance between the Ti disk array and Al_2_O_3_. 

**Figure 4:**
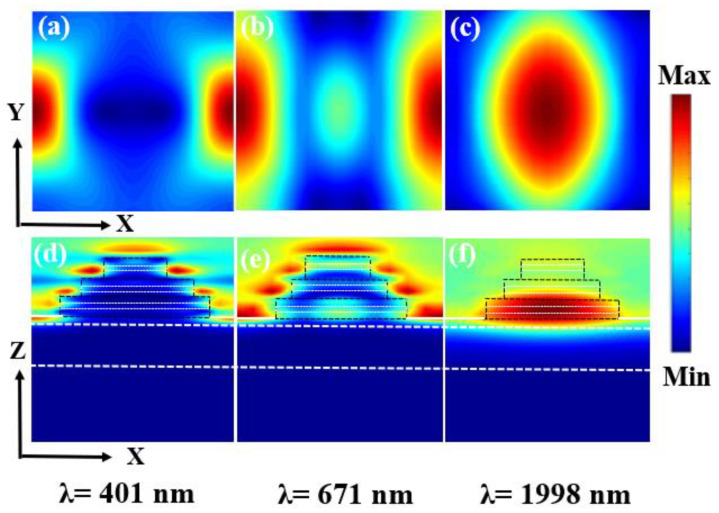
(**a**–**c**) is the intensity of the electromagnetic field distributed in one period at different resonant wavelengths in the XOY plane. (**d**–**f**) is the intensity of the electromagnetic field distributed in one period at different resonant wavelengths in the XOZ plane. The wavelengths are: 401 nm, 671 nm, 1998 nm.

In Figure 4, we also show the distribution of the magnetic field strength within the cell structure for incident light with wavelengths of 401 nm, 671 nm, and 1998 nm to further demonstrate the mechanism of broadband absorption. According to Figure 4, when the incident wavelength is not large, on the edge of the composite component structure layer and in several adjacent composite layers are where the magnetic field is mostly found. The magnetic field gradually grows at the composite layer’s edge with increasing incident wavelength and is primarily distributed in numerous nearby composite element constructions at the bottom. Because the composite layers of Ti and Al_2_O_3_ form the MIM structure, it is the physical basis of the exciton [47]. Moreover, in this unit structure, the radius of the disc of the composite layer decreases gradually from the bottom to the top, and the exciton excitation wavelength is connected with the radius of the disc of the composite layer in the unit structure. Therefore, as the wavelength increases, the position of the exciton in the unit structure gradually shifts downward. The multiple disc radii of the composite layer correspond to the multiple excitation wavelengths of the exciton, so that broadband absorption can be achieved.

**Figure 5:**
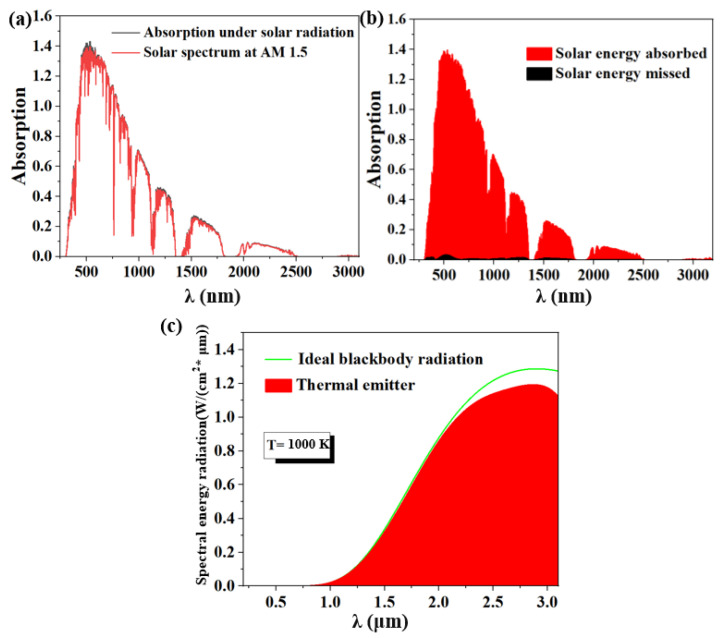
(**a**,**b**) Curves of the absorber’s solar energy absorption and loss from 280 to 3100 nm at air mass (AM) 1.5. (**c**) Thermal emission.

A key parameter to evaluate the heat resistance of ideal absorbers is full spectrum absorption. By putting the absorber in air, we attempt to determine the whole spectral absorption rate (AM1.5). The mass of the perfect absorber is 1.5 at 1000 K, the black is the lost energy, and the red is the absorbed energy. According to Figure 5b, the energy spectra of the solar absorber are shown in red, while the energy spectra of the 1.5 air mass are shown in black. In the 280 nm to 3100 nm region, the weighted average absorption efficiency is up to 98.3%, and the loss is incredibly tiny. The experimental results demonstrated a moderate absorption rate over the whole spectrum for the proposed solar absorber. Figure 5c describes the solar absorption system’s exothermic characteristics at a high temperature of 1000 K. According to Kirchhoff’s law, the thermal emission ε(ω) is equivalent to the absorption A(ω). Since the transmittance of the opaque metal is equal to zero, the spectrum ε(ω) can be related to the following law, ε(ω) = 1 − R(ω). For thermal emission based on Planck’s law, the thermal emissivity (η_E_) can be expressed as Equation (2) [48,49]:

(2) $$\eta_{E}=\frac{\int_{\lambda_{\min}}^{\lambda_{\max}}\varepsilon \left(\omega \right)\cdot I_{\mathrm{be}}\left(\omega ,T\right)d\omega }{\int_{\lambda_{\min}}^{\lambda_{\max}}I_{\mathrm{be}}\left(\omega ,T\right)d\omega }$$

The ideal blackbody spectrum’s intensity at frequency ω and temperature T is represented by the notation I_be_(ω, T). As observed in Figure 5c, the emitter’s emission intensity is nearly perfect and its emission efficiency is higher than that of the ideal blackbody model in the wavelength range of 2000 nm. This indicates that a refractory-based blackbody emitter or light source is feasible and substantial.

In this paper, six different structures of solar absorbers were studied, their spectral absorption properties were compared, and the optimal absorption spectra were obtained. The fire-resistant solar absorber has multiple resonance points in the range of 200–3100 nm and superimposed on each other to form multiple absorption peaks. Therefore, the average absorbance of the absorber [50] can be defined as

(3) $$A_{\mathrm{aver}}=\frac{\int_{\lambda_{\min}}^{\lambda_{\max}}A\left(\lambda \right)d\lambda }{\lambda_{\max}-\lambda_{\min}}$$

where A is the absorbance at that wavelength, and the maximum and minimum values of the incident wavelength are expressed as λ_max_ and λ_min_, respectively. According to the above equation, the average absorbance of the metamaterial solar absorber in the 200–3100 nm band is 95.8%.

**Figure 6:**
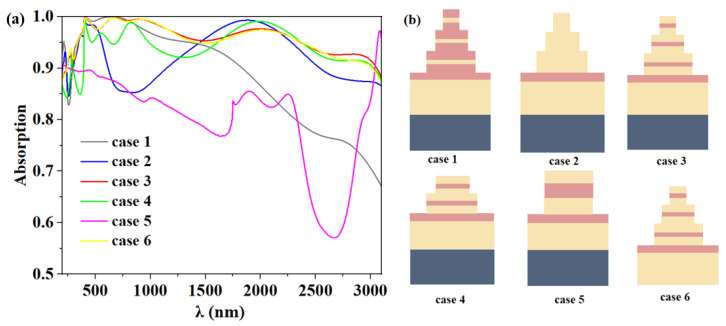
(**a**) A variety of micro/nanostructures’ absorption spectra. (**b**) Model diagrams corresponding to six different structures.

The computed average absorption efficiency and the absorption spectra of the absorbers with various configurations are presented in Figure 6a,b, respectively, to highlight the advantages of the proposed absorber structure. As shown in Figure 6a, it operates well in the near-UV and visible bands when the MIM structure of the top layer of the absorber is switched out for an IMI grating structure, but it gradually degrades in the near-IR band and all the mid-IR bands, with an average absorption efficiency of 88.4%. When the upper stacking discs of the absorber were all of the same material Ti, the absorption in the near-infrared band dropped to below 90%. Even though it increased subsequently, the mid-infrared band’s average absorption fell to 92.3%, dropping below 90% once more. The UV band absorption is lower, below 90%, with an average bandwidth absorption of 94.2% when the absorber only contains the first and second layers of the composite disk structure. The absorption in the entire band is almost less than 90%, particularly in the mid-infrared band, when the MIM structure of the upper layer of the absorber is replaced by a composite disk structure of the same radius and thickness [51]. When the bottom structure of the absorber has no substrate W, the overall absorption performs well, but absorption rates in the mid-infrared band start to drop below 90%, which limits the absorption in the ultra-wideband absorption. Comparatively, the results showed that the solar absorber based on the three-layer MIM stacked structure had an average absorption efficiency of 95.8%, good overall absorption efficiency, with the largest absorption band being 2811 nm.

**Figure 7:**
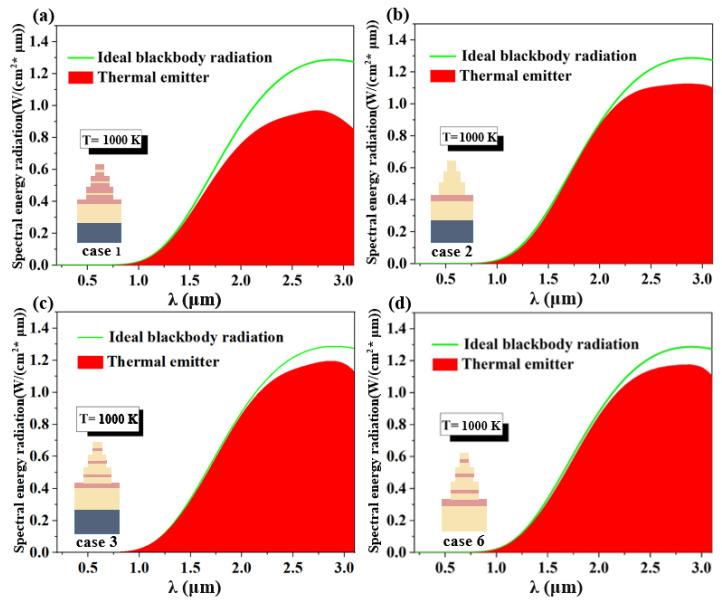
(**a**–**d**) Thermal radiation pattern of case 1, 2, 3, 6 at 1000 K.

Additionally, we contrasted the thermal emission effectiveness of several models at 1000 K. Case 5’s overall absorption rate was too low, so we immediately dismissed it. Case 4’s absorption rate in the UV band was likewise too low, restricting the broadband range of our absorber, so it is also not considered. Therefore, as shown in Figure 7, we compared the thermal emission plots of the four models for Cases 1, 2, 3, and 6. As seen in Figure 7a, Case 1’s thermal emission efficiency significantly declines from the near-infrared region, with the average thermal emission efficiency for the entire band being just 80.5%. In Figure 7b, Case 2 has good overall thermal emission efficiency, and the average thermal emission efficiency in the whole band is 92.4%, but it is still slightly poor in the mid-infrared band. The thermal emission efficiency of Case 3 proposed in this paper is stronger and nearly perfect; as shown in Figure 7c, the average emission efficiency in the whole band is as high as 94.4%. We then observe Figure 7d for Case 6 in which the structure of removing the substrate W, the average thermal radiation efficiency is 93.9%, the overall thermal radiation efficiency does not change much, but the maximum radiation efficiency and average thermal emission efficiency are lower than Case 3, which is slightly inferior. We ultimately decided that Case 3 is the best structure, serving as the ultra-broadband ideal absorber and thermal emitter in this study, after taking into account the absorption efficiency, broadband absorption, and thermal emission efficiency of the aforementioned various designs [52,53]. 

**Figure 8:**
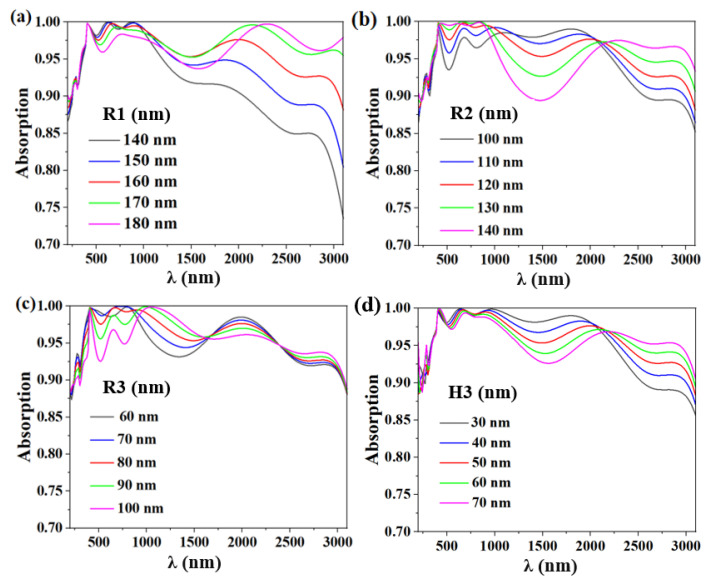
(**a**) The absorption efficiency of disk R1 at 140–180 nm. (**b**) The absorption efficiency of disk-shaped R2 at 100–140 nm. (**c**) The absorption efficiency of disk R3 at 60–100 nm. (**d**) The absorption pattern of Al_2_O_3_ film thickness H3 at 30–70 nm.

Several main structural parameters affecting the absorption rate are discussed. When the first nanodisk’s radius is R1, as shown in Figure 8a, the absorption efficiency increases from 140 nm to 180 nm. The absorption effectiveness is shown in Figure 8b when the second nanodisk’s radius R2 grows from 100 nm to 140 nm. The third layer composite structure’s absorption effectiveness, with radius R3 in the range of 60 nm to 100 nm, as shown in Figure 8c. As can be seen in the figure, it is discovered that increasing the radius has a more pronounced impact on the near-infrared band while having less of an impact on the near-UV-visible band. We take into account how the nanodisk’s radius affects the effectiveness of its absorption [54,55,56]. The absorption curve for the change in nanodisk radius is shown in Figure 8a–c. When the radius of the nanodisk is small, the high interstructure plasma spacing, small size, and weak near-field coupling effect result in a narrow absorption band and a weak broad absorption strength. The near-field coupling effect between the structures strengthens as the radius rises, increasing the broadband absorption of the spectrum similarly to how the average absorption efficiency does [57,58,59]. However, the absorption effect in the 200–2000 nm band becomes worse as the radius increases. Thus, after taking into account the overall absorption efficiency, we arrived at the final radius values of R1 = 160 nm, R2 = 120 nm, and R3 = 80 nm.

The figure also demonstrates that altering the Al_2_O_3_ nanofilm’s thickness can change the bandwidth range of the absorber, offering a fresh approach to modifying the broad frequency range of the absorption spectrum and enhancing the band’s overall absorption efficiency. The near-infrared absorption rate increases with nanosheet thickness between the wavelengths of 500 nm and 1200 nm, and its absorption efficiency approaches 100%. The absorptivity in mid-infrared region decreases with the increase in thickness. After comprehensive consideration, the final parameter H3 = 50 nm was selected to obtain the best possible absorption efficiency. The final values are R1 = 160 nm, R2 = 120 nm, R3 = 80 nm, and H3 = 50 nm for the dielectric layer. We ultimately conclude that the period is P = 400 nm since the structure period has a similar impact on the absorption outcome as the radius and because the plasma’s near-field effect [60] also affects the final absorption effect.

**Figure 9:**
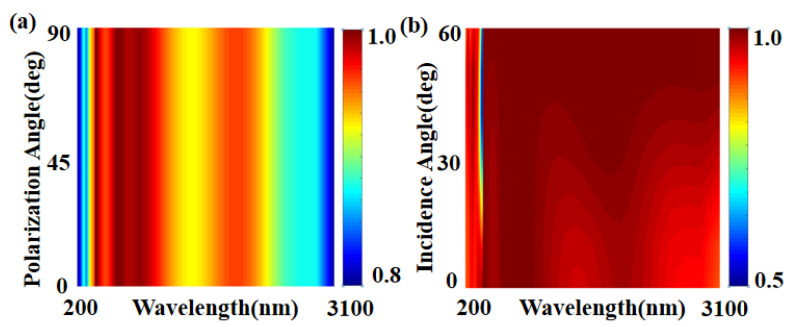
(**a**) The absorption spectra for polarization angles between 0° and 90°. (**b**) The absorption spectrum for varying incidence angles of 0° to 60°.

We know that in a real environment, natural light cannot be vertically incident on a solar absorber, as it would be in an ideal situation [61,62,63,64]. Therefore, it is important to talk about how different polarization and incidence angles affect the performance of solar absorbers. As shown in Figure 9, we simulated the absorption spectra using incidence angles between 0° and 60° and polarization angles between 0° and 90°, respectively. As can be observed from the figure, the produced absorber has an excellent effect with extraordinary absorption efficiency in the incidence angle range of 0° to 60° and has no effect on the change in incidence angle. Because of the high geometric symmetry of the structure, the absorptivity remains high over the whole wavelength range for the polarization Angle [65,66,67]. With stronger oblique incidence and polarization insensitivity, the absorber’s performance has generally increased greatly [68].

## 4. Conclusions

A metal-dielectric-metal composite structure was used in the design of our ultra-wideband solar absorber. It is composed of W-Ti-Al_2_O_3_ film composite structure and three Ti-Al_2_O_3_-Ti disks with different radii. We make use of the refractory substance W, which melts at 3420 °C, and the refractory metal Ti, which melts at 1668 °C. Because the dielectric layer Al_2_O_3_ is also a high-temperature insoluble substance with a melting point of 2054 °C, the structure works without any influence at high temperatures. The structure achieves an almost perfect absorption bandwidth of 2811 nm (244–3055 nm) with an average absorption efficiency of 95.8% in this range when taking into account the absorption rate and bandwidth. The main reason for achieving this ultra-wideband absorption characteristic is the synergistic effect of guided-mode resonance, surface shaping, and cavity mode in this wavelength range. Additionally, according to the air mass absorption spectrum equation (AM1.5) and the blackbody radiation calculation, our absorber’s weighted average absorption efficiency in the range of 280–3100 nm is 98.3%, and the solar energy loss is only 1.7%. At 1000 K, the emission efficiency is 94.4%. The structure has good absorption and thermal radiation properties, and exhibits strong polarization angle and incident angle insensitivity. In addition, this ultra-wideband absorption property is highly plastic in terms of structural period, thickness of each layer, disc radius, and metallic material, and has great potential for thermal energy harvesting, thermoelectronic components, and optical imaging.

## Figures and Tables

**Table 1:** Performance comparison between different absorber designs proposed in previous publications.

Reference	Construction	A Range of Wavelengths with Absorbance Greater than 90%	Absorption Effectiveness on Average	The Average AM1.5 Absorption Efficiency
[40]	TiN disc-square ring resonator	2200 nm	94.0%	89.0%
[41]	Ti-SiO_2_-Ti	1650 nm	(250–3000 nm)	(250–3000 nm)
[42]	A layered elliptic structure	1868 nm	93.3%	88.2%
[43]	Two-dimensional colloidal arrays and semiconductor germanium voids	1100 nm	(295–2500 nm)	(295–2500 nm)
[44]	Bihexagonal metamaterial and Si ring column structure	1200 nm	90.0%	88.0%
Proposed	Two Ni disk structures of different sizes are used	2811 nm	(250–3500 nm)	(250–4000 nm)

## Data Availability

Publicly available datasets were analyzed in this study. This data can be found here: [https://www.lumerical.com/ (accessed on 1 January 2020)].
